# Chronic cannabis promotes pro-hallucinogenic signaling of 5-HT2A receptors through Akt/mTOR pathway

**DOI:** 10.1038/s41386-018-0076-y

**Published:** 2018-04-27

**Authors:** Inés Ibarra-Lecue, Irene Mollinedo-Gajate, J Javier Meana, Luis F Callado, Rebeca Diez-Alarcia, Leyre Urigüen

**Affiliations:** 1 Department of Pharmacology, University of the Basque Country UPV/EHU and Centro de Investigación Biomédica en Red de Salud Mental CIBERSAM, Leioa, Spain; 2 Biocruces Health Research Institute, Bizkaia, Spain

## Abstract

Long-term use of potent cannabis during adolescence increases the risk of developing schizophrenia later in life, but to date, the mechanisms involved remain unknown. Several findings suggest that the functional selectivity of serotonin 2A receptor (5-HT2AR) through inhibitory G-proteins is involved in the molecular mechanisms responsible for psychotic symptoms. Moreover, this receptor is dysregulated in the frontal cortex of schizophrenia patients. In this context, studies involving cannabis exposure and 5-HT2AR are scarce. Here, we tested in mice the effect of an early chronic Δ^9^-tetrahydrocannabinol (THC) exposure on cortical 5-HT2AR expression, as well as on its in vivo and in vitro functionality. Long-term exposure to THC induced a pro-hallucinogenic molecular conformation of the 5-HT2AR and exacerbated schizophrenia-like responses, such as prepulse inhibition disruption. Supersensitive coupling of 5-HT2AR toward inhibitory Gαi1-, Gαi3-, Gαo-, and Gαz-proteins after chronic THC exposure was observed, without changes in the canonical Gαq/11-protein pathway. In addition, we found that inhibition of Akt/mTOR pathway by rapamycin blocks the changes in 5-HT2AR signaling pattern and the supersensitivity to schizophrenia-like effects induced by chronic THC. The present study provides the first evidence of a mechanistic explanation for the relationship between chronic cannabis exposure in early life and increased risk of developing psychosis-like behaviors in adulthood.

## Introduction

Cannabis consumption especially in early adolescence [1], a period of increased vulnerability to its effects, increases the risk of developing schizophrenia [2]. Both cannabis extracts and Δ^9^-tetrahydrocannabinol (THC) can evoke transient psychotic states in healthy subjects [3] and worsen symptoms in schizophrenia patients [4]. However, the neuronal and molecular mechanisms underlying psychotic symptoms elicited by cannabis abuse during adolescence remain unknown.

Several findings suggest that serotonin 2A receptors (5-HT2AR) are involved in the molecular mechanisms responsible for psychotic symptoms. In addition to the canonical signaling pathway through Gαq/11-proteins, hallucinogenic 5-HT2AR agonists, such as lysergic acid diethylamide (LSD) and 2,5-dimethoxy-4-iodoamphetamine ((±)-DOI), which trigger mental states resembling psychotic symptoms [5] activate inhibitory Gαi/o proteins [6]. Conversely, non-hallucinogenic 5-HT2AR agonists exclusively activate the canonical signaling pathway through Gαq/11-proteins. This differential signaling mechanism responsible for the unique effects of hallucinogens is known as biased agonism.

The disruption of 5-HT2AR functionality in psychosis is supported by genetic studies describing differential epigenetic methylation and gene polymorphisms in subjects with schizophrenia [7]. Animal models of schizophrenia have shown increased 5-HT2AR density and/or functionality [8, 9]. Moreover, it has been demonstrated that the active conformation of the 5-HT2AR is up-regulated in prefrontal cortex of antipsychotic-free schizophrenia subjects [10]. Furthermore, atypical antipsychotics display high affinity as antagonists of 5-HT2AR. All these data demonstrate that upregulation and/or increased functionality of 5-HT2AR could predispose to psychosis or schizophrenia.

Akt/mTOR signaling pathway mediates several functions, including synaptic plasticity and axonal branching [11]. Akt protein dysregulation has been linked to schizophrenia [12, 13], and, in addition, *AKT1* genetic variations have been associated with increased psychotic symptoms after smoking cannabis [14]. Importantly, acute THC activates this signaling pathway in mouse brain [15].

Considering these findings, we investigated whether chronic THC exposure at young ages could lead to an over-functionality of brain cortical 5-HT2AR with biased agonism toward pro-hallucinogenic G-protein pathways. In addition, the possible involvement of Akt/mTOR signaling pathway in the induction of this psychosis-like status was studied.

## Materials and methods

### Animals

All experimental procedures were performed in accordance with the European Directive for the Protection of Vertebrate Animals used for experimental and Other Scientific Purposes (European Union Directive 2010/63/EU) and approved by the Ethic Committee for Animal Welfare of the University of the Basque Country (UPV/EHU) (CEBA 270M/2012, 189/2011). Male CD-1 mice (Charles Rivers, Wilmington, MA, USA) were housed (6–8 animals per cage) under controlled temperature (23 ± 1 °C), on a normal 12 h light/dark cycle, with free access to food and water. Only male mice were used in this study to avoid the effects of the female estrous cycles that can interfere with the pharmacological parameters.

### Treatments

THC, rapamycin and vehicle were administered for 30 days starting at postnatal day 21. Drugs were dissolved in ethanol:cremophor:saline (0.9% NaCl) at 1:1:18 ratio. THC (THCPharm. GmbH. Frankfurt, Germany) (10 mg/kg ip) [15] was injected daily. Rapamycin (Shelleck Chemicals LLC, Houston, TX, USA) (5 mg/kg ip) was administered in a 1 day on/1 day off schedule [16], 30 min before THC injection. For prepulse inhibition (PPI) experiments, (±)-DOI (Sigma-Aldrich, St. Louis, MO, USA) (0.5 mg/kg ip) [17] was dissolved in saline and administered immediately before the behavioral assay. Experiments were carried out after a 5-day washout period to avoid the interference of acute effects of THC.

### Experimental design

Experimental design of each assay was conducted as follows. A first batch of two groups of animals (vehicle and THC, *n* = 16 animals per group) was treated with THC or vehicle for 30 days. After a 5-day washout period, a number of animals (*n* = 8 per group, vehicle and THC) were killed, brains removed and THC concentrations measured. The rest of the animals (*n* = 8 per group, vehicle and THC) were used to perform basal PPI, 5-HT2AR density, radioligand binding and functional coupling experiments (*n* = 8 per group, pooled for in vitro assays). Briefly, after the 5-day washout period, PPI experiments were carried out and later, animals were killed, brains removed, and in vitro assays performed.

Another different batch of four groups of animals (vehicle-vehicle, vehicle-THC, rapamycin-vehicle and rapamycin-THC) (*n* = 8 per group) was stablished to carry out basal PPI, western blot experiments (Akt, p-Akt, rpS6, p-rpS6) and functional coupling experiments (*n* = 8 per group, pooled for in vitro assays) following these different pharmacological treatments.

Finally, another batch of four groups (vehicle-vehicle, vehicle-THC, rapamycin-vehicle and rapamycin-THC) was used to accomplish PPI experiments after acute DOI in a timely matched manner (*n* = 8 per group).

### Quantification of THC concentration in mouse brain by liquid chromatography–tandem mass spectrometry

Pooled homogenized THC-free mouse brain samples (*n* = 6–8) were used for the development and validation. Experiments were carried out with a 1290 Infinity II ultrahigh performance liquid chromatography (UHPLC) system coupled to a 6495 iFunnel triple Quad mass spectrometer equipped with a JetStream electrospray ionization source (Agilent Technologies, Santa Clara, CA, USA). A Kinetex EVOC18 100A 3 × 100 mm (2.6 μm) column was selected. Sample analysis in positive ionization mode was performed using as mobile phase water with 0.1% formic acid (solvent A) and acetonitrile with 0.1% formic acid (solvent B) with elution gradient mode. The flow rate was 0.5 ml/min; injection volume was 5 μl, and the column temperature was maintained at 30 °C. The calibration curve was prepared by spiking 20 μl of standard working solution to obtain brain THC final concentrations of 8–2000 ng/g. Quality control samples (QCs) were prepared by spiking brain samples containing 60 and 1000 ng/g as the final THC concentration.

### Prepulse inhibition of the startle reflex

Prepulse inhibition (PPI) test was performed in a startle chamber (PanLab, Barcelona, Spain) where a 60-dB background-noise was present for a 10 min acclimatization period, as well as throughout the entire experiment. Each session began with five startle pulse-alone trials to achieve a more stable response of the animals and were not included in the analysis. Mice were then subjected to a pseudo-randomized combination of: ten pulse-alone trials consisted of a white noise burst (120 dB, 40 ms); ten prepulse-alone trials for each prepulse intensity (10 ms at 77, 82, or 87 dB); ten prepulse-pulse trials for each prepulse intensity with an interval of 60 ms between both and ten no-stimulus trials, in which only the background noise was present. The inter-trial intervals were 10, 12, 15, 20, and 25 s. The maximum amplitude of the startle reaction was recorded for every trial, and the average startle for each trial subgroup was used for the analysis. No-stimulus trials and prepulse-alone trials did not exert any startle response and data were not included in the analysis. PPI was calculated as a percentage score: % PPI = 100—[(startle response to prepulse-pulse trial/startle response to pulse-alone trial) × 100]. Inhibition of the PPI response was induced by administration of the 5-HT2AR agonist (±)-DOI (0.5 mg/kg, i.p.) immediately before acclimation period. Each session lasted 30–33 min.

### Brain cortex membranes preparation

Animals were killed by cervical dislocation, brains removed, cortex dissected, and samples stored at −80 °C until use. Membrane-enriched fraction (P2) was prepared as described [18].

### Radioligand binding

[^3^H]Ketanserin saturation binding assays were performed as previously reported [19] with minor modifications. [^3^H]Ketanserin binding (0.05–10 nM; eight concentrations) was used to calculate density (Bmax) and affinity (Kd) of 5-HT2AR. Non-specific binding was determined in the presence of the 5-HT2AR antagonist M100907 (1 μM). Competition curves of [^3^H]ketanserin binding (2 nM) with increasing concentrations of the agonist (±)-DOI (10^−12^–10^−3^ M) were also performed in order to delineate both the G-protein coupled and uncoupled 5-HT2AR conformations. Briefly, after incubation (60 min, 37 °C), free radioligand was discarded by rapid filtration under vacuum (1450 FilterMate Harvester, Perkin Elmer, Waltham, MA, USA). Filters were then rinsed, dried and bagged in Sample Bag with BetaPlate Scint scintillation cocktail. Radioactivity was detected by liquid scintillation spectrometry using a MicroBeta TriLux counter (Perkin Elmer, Waltham, MA, USA).

### Antibody-capture [^35^S]GTPγS scintillation proximity assay (SPA)

Specific activation of different subtypes of Gα-proteins by (±)-DOI (10 µM) was determined using a previously described protocol [18]. The concentration of (±)-DOI (10 µM) was chosen for being the one which induces stimulation values around the Emax for any Gα subunit subtype under our experimental conditions [19]. [^35^S]GTPγS binding was performed in buffer containing 0.4 nM [^35^S]GTPγS, 15 μg of protein/well and different GDP concentrations depending on the Gα-subtype tested. Specific antibody for each Gα-subunit and polyvinyltoluene SPA beads coated with protein-A were added and incubated (3 h, room temperature). Plates were centrifuged and bound radioactivity detected on a MicroBeta TriLux scintillation counter. Non-specific binding was defined as the remaining [^35^S]GTPγS binding in the presence of 100 μM unlabeled GTPγS. The antagonist ketanserin (10 μM) was used to confirm the 5-HT2AR involvement in the observed stimulations (data not shown).

### Western blot

Western blot experiments were performed with total homogenate fractions (S1) from mouse brain cortex tissue. Samples (30 μg) were heated (95 °C), loaded onto polyacrylamide gel (12%) and submitted to SDS-PAGE. Nitrocellulose membranes were blocked (5% non-fat dry-milk or/and 0.5% BSA) in TBS buffer followed by overnight incubation with primary antibodies (4 °C). Antibodies against 5-HT2AR, Akt, phospho-Akt(Ser473), rpS6, phospho-rpS6(Ser235/236) and β-actin were used. Incubation with fluorescent anti-IgG secondary antibodies was performed at room temperature (1 h). Immunoreactivity was quantified using an Odyssey Infrared Imaging System (LI-COR Biosciences, Lincoln, NE, USA) (further details in Supplementary Table 1).

### Data analysis and statistical procedures

Data were analyzed with GraphPad Prism™ 5.01, and InVivoStat software. Before the statistical analyses, the data were inspected for outliers (critical value, *Z* > 1.96) using Grubb’s test (GraphPad Software, www.graphpad.com/quickcalcs/grubbs1.cfm). In PPI experiments, data from animals (between 0 and 3 animals per group) showing an outlier value for a particular dB or for the startle amplitude to the pulse were discarded for further analysis. Two animals were also discarded for not showing any response to the startle stimulus. Two-group comparisons were made by unpaired Student’s *t*-test. Multiple groups’ comparisons were studied by one-way, two-way or three-way analysis of variance (ANOVA), followed by Bonferroni’s or Benjamini-Hochberg’s post hoc analyses. Statistical significance was set at *p* < 0.05. Displacement curves were analyzed using a nonlinear fit, and the selection between models was made by the extrasum-of-squares (*F*-test). Following the nonlinear curve fitting, Ki values for (±)-DOI were calculated from the corresponding IC50 values. Differences in the binding profiles were assessed by unpaired Student’s *t*-test of normalized (log) parameters. Specific binding values obtained from SPA assays were transformed to percentage of basal binding (binding values observed without agonist drug) obtained for each Gα-protein and analyzed by one-sample or two-sample Student’s *t*-test. The immunodensitometric values of the different target proteins were normalized to the intra-assay values obtained with anti-β-actin antibody and expressed as mean ± SEM of the percentages over an inter-assay normalization sample included in every experiment. Each sample was analyzed at least in two independent experiments.

## Results

### Time-dependent concentration of THC in mouse brain cortex

**Fig. 1:**
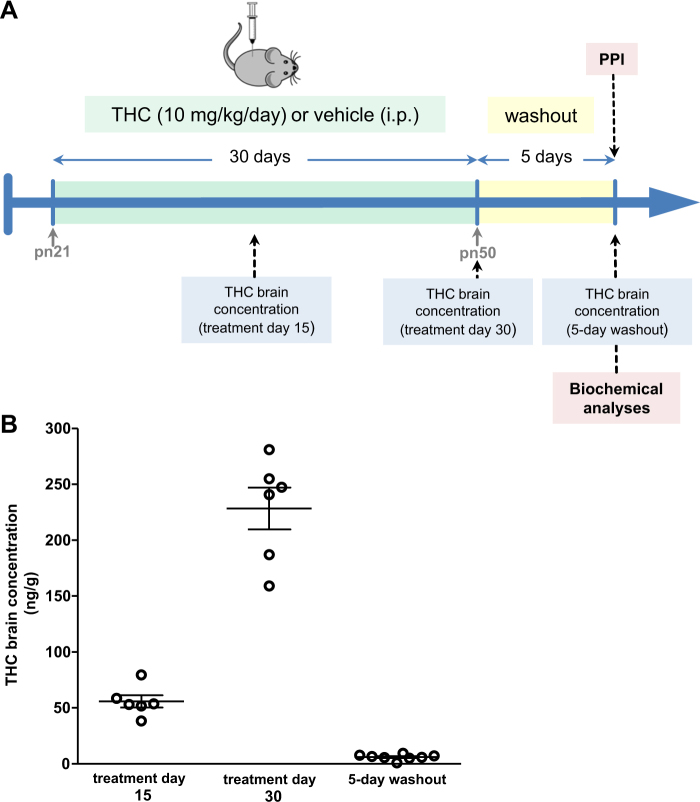
Chronic THC treatment protocol and concentration. **a** Representation of the treatment design. **b** THC concentrations in mouse brain (*n* = 6–8) during treatment and after the washout period. Points represent individual data for each animal and lines represent means

In order to validate the chronic administration protocol, brain THC concentrations were measured at treatment days 15 and 30, and after a 5-day washout period (Fig. 1a). THC reached 56 ng/g after 15 days of daily-chronic treatment. This concentration increased fourfold at day 30 (228 ng/g), while brain THC levels largely disappeared (6.05 ng/g) after the washout period (Fig. 1b).

### Chronic THC potentiates (±)-DOI-induced decrease in PPI

**Fig. 2:**
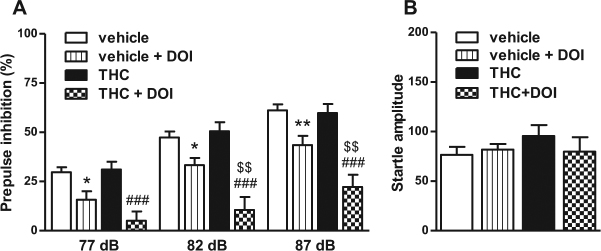
Chronic THC increases the disruption of PPI induced by the 5-HT2AR agonist (±)-DOI. **a** Chronic THC exacerbated the (±)-DOI-induced PPI disruption (three-way repeated measures ANOVA: THC × (±)-DOI; *F*(1,51) = 6.48, *p* < 0.05; *n* = 8–16). Benjamini-Hochberg’s post hoc comparisons: *p < 0.05, **p < 0.01 vs. vehicle. ###*p* < 0.001 vs. THC, $$*p* < 0.01 vs. vehicle-DOI (see Supplementary Table 3 for further details on ANOVAs). **b** No differences were found in the startle amplitude among groups. Bars represent mean ± SEM

Loss of PPI in rodents is considered a valid proxy for the study of the neurobiology of impaired sensorimotor gating in schizophrenia patients [20, 21]. Disruptions of the PPI response by acute administration of the 5-HT2AR agonist (±)-DOI were evaluated in vehicle and THC-treated animals. We used a dose of 0.5 mg/kg (±)-DOI that has demonstrated, under our experimental conditions, to induce a significantly decrease in the PPI (Supplementary Figure 1). Chronic THC significantly exacerbated the (±)-DOI-induced disruption of PPI (Fig. 2a). Chronic THC alone did not alter basal PPI, similar to previous findings [22]. No significant differences among groups were found in the amplitudes of the startle reflex (Fig. 2b).

### Chronic THC does not alter total 5-HT2AR density in brain cortex

**Fig. 3:**
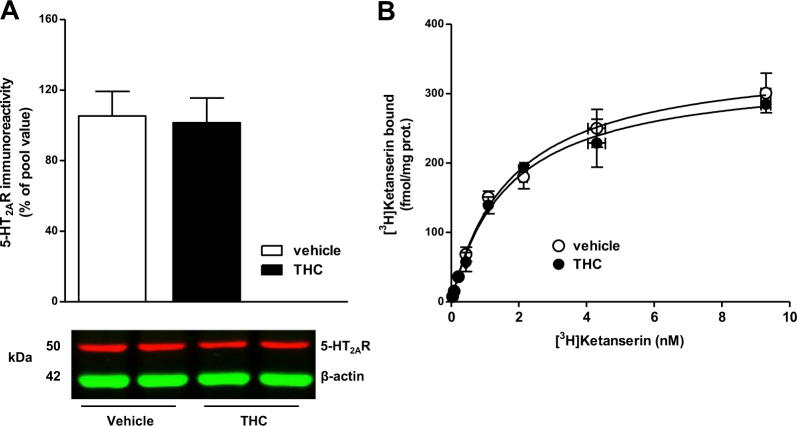
Chronic THC does not modulate 5-HT2AR protein density. **a** 5-HT2AR immunodensity in brain total homogenates of vehicle- and THC-treated mice (*n* = 8 each group). Representative immunoblot of 5-HT2AR and β-actin in vehicle and THC-treated mice. **b** Specific [^3^H]ketanserin binding saturation curves in cortical membranes of vehicle- and THC-treated mice (pool of *n* = 8 each group). Bars and points represent mean ± SEM

Next, we investigated the effects of chronic THC on cortical 5-HT2AR, the main molecular target of (±)-DOI. Protein immunodetection of 5-HT2AR showed a single band at ~53 kDa, as previously described [23]. Chronic THC did not alter 5-HT2AR protein expression (Fig. 3a). In order to confirm this result, saturation binding experiments with the 5-HT2AR antagonist [3H]ketanserin were performed in brain cortical membranes. No significant differences in density (~340 fmol/mg protein) were observed between both groups (Fig. 3b).

### Chronic THC modulates the functional coupling of cortical 5-HT2AR

**Fig. 4:**
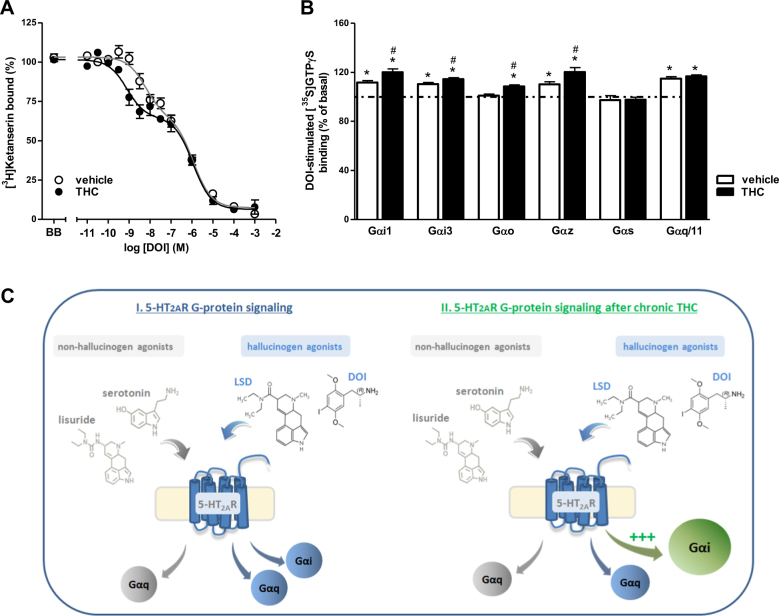
Chronic THC promotes 5-HT2AR signaling through inhibitory G-proteins. **a** Competition curve of [^3^H]ketanserin specific binding (2 nM) by (±)-DOI in brain membranes of vehicle- and THC-treated mice (pool of *n* = 8 each group). In both cases, data were best fit to a biphasic compared to monophasic displacement curve (*F*-test, *p* < 0.0001, for both curves). (±)-DOI showed supersensitivity for the high-affinity subpopulation of 5-HT2AR (**p < 0.01) (see Supplementary Table 2 for details) after chronic THC. **b** (±)-DOI (10 μM) induced Gαi1-, Gαi3-, Gαz-, and Gαq/11-protein activation (*p < 0.05 vs. basal) in brain cortical membranes of vehicle-treated mice (pool of *n* = 8 each group). Chronic THC elicited a supersensitivity for (±)-DOI-induced Gαi1-, Gαi3-, Gαo-, and Gαz protein activation (#*p* < 0.05 vs. vehicle). Points and bars represent mean ± SEM of 4–6 different experiments carried out in triplicate. **c** Suggested model of the 5-HT2AR signaling modulation after chronic THC

In order to deeper evaluate the molecular conformation of the 5-HT2AR, we examined the pharmacological parameters of (±)-DOI displacing [^3^H]ketanserin binding in brain cortex membranes. The agonist (±)-DOI displaced the antagonist [^3^H]ketanserin binding in a biphasic manner (Fig. 4a). Comparing both the high- and low-affinity binding populations dissected by the agonist in vehicle- and THC-treated animals, the affinity of (±)-DOI for the high-affinity 5-HT2AR population was significantly increased after chronic THC. No differences were found in any other pharmacological parameter (Supplementary Table 2).

We next evaluated the functionality of 5-HT2AR by studying the agonist-induced functional coupling to different Gα-protein subtypes. The agonist (±)-DOI produced a selective activation of Gαi1-, Gαi3-, Gαz-, and Gαq/11-proteins but not of Gαo- and Gαs-proteins in vehicle-treated mice. The activation of all these Gα-proteins was antagonized in the presence of ketanserin (10 μM) (data not shown). The Gαq/11-protein stimulation showed no significant differences between groups. By contrast, after THC chronic treatment, stimulation of Gαi1-, Gαi3-, Gαo-, and Gαz-proteins by (±)-DOI was significantly increased. Stimulation of Gαo-protein was only observed in THC-treated mice (Fig. 4b). Therefore, chronic THC exposure induced an overstimulation of inhibitory G-protein-mediated signaling (Fig. 4c).

### Akt/mTOR signaling pathway is involved in the sensitization of 5-HT2AR induced by chronic THC

**Fig. 5:**
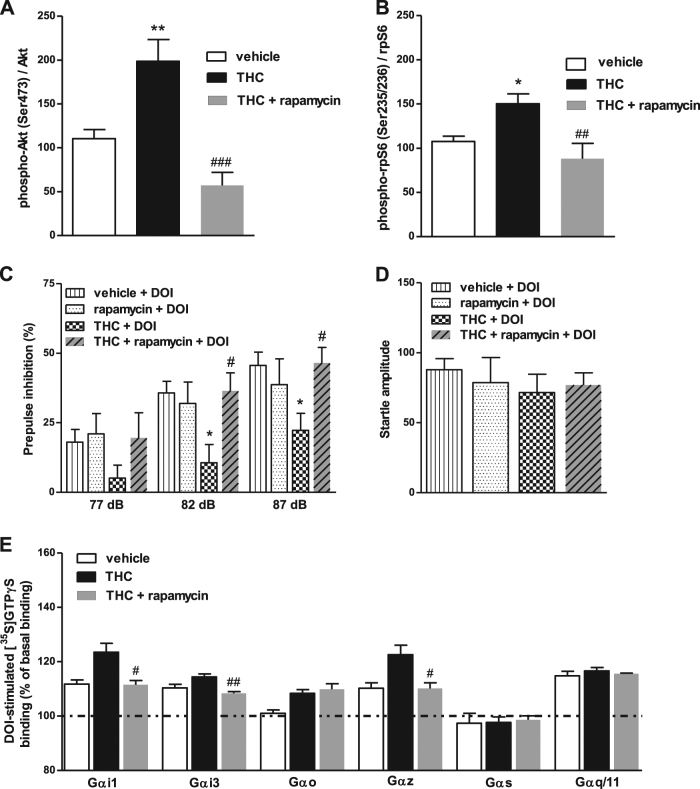
Akt/mTOR signaling pathway is involved in the THC-induced sensitization of 5-HT2AR signaling. Chronic THC increased phosphorylation of (**a**) Akt and (**b**) rpS6, and concomitant treatment with mTORC1 inhibitor rapamycin blocked these effects (one-way ANOVA: phospho-Akt/Akt ratio *F*(2,20) = 15.68, *p* < 0.0001; phospho-rpS6/rpS6 ratio *F*(2,16) = 8.04, *p* < 0.01; *n* = 5–8). Bonferroni’s post hoc comparisons: *p < 0.05, **p < 0.01 vs. vehicle. ##*p* < 0.01, ###*p* < 0.001 vs. THC. **c** Rapamycin blocked the sensitization to (±)-DOI effect on PPI in THC-treated mice, restoring it to vehicle-values among all the prepulses (three-way repeated measures ANOVA: THC × rapamycin; *F*(1,35) = 4.50, *p* < 0.05; *n* = 8–16). Benjamini-Hochberg’s post hoc comparisons: *p < 0.05 vs. vehicle + DOI. #*p* < 0.05 vs. THC + DOI (see Supplementary Table 3 for further details on ANOVAs). Data of vehicle + DOI and THC + DOI groups are also in Fig. 2a. **d** No changes were found in the startle amplitude among groups. Data of vehicle + DOI and THC + DOI groups are also in Fig. 2b. **e** Rapamycin blocked the (±)-DOI-induced hyperactivation of Gαi1-, Gαi3-, and Gαz- (#*p* < 0.05, ##*p* < 0.01 vs. THC), but not that of Gαo proteins (pool of *n* = 8 each group). Bars represent mean ± SEM of 4–6 different experiments carried out in triplicate

In order to tackle the putative role of Akt/mTOR pathway in this functional modulation, mice were treated with both THC and the mTOR inhibitor rapamycin. Chronic THC significantly activated (increase of phosphorylated forms) immunoreactivity of both Akt and ribosomal protein S6 (rpS6), a downstream effector of mTOR, in cortical tissue. Moreover, this activation was abolished in the presence of rapamycin (Fig. 5a, b). Rapamycin also blocked the effect of THC treatment in the PPI response to (±)-DOI and restored it to vehicle-values (Fig. 5c), without changing the startle response (Fig. 5d). Finally, rapamycin treatment fully blocked the THC-induced hyperactivation of Gαi1-, Gαi3-, and Gαz-proteins by (±)-DOI, but not that of Gαo-protein (Fig. 5e). For the effects of chronic rapamycin alone, see Supplementary Figures 2-4.

## Discussion

Several pharmacological and behavioral effects of THC have been well documented in animal models, although little is known about the mechanism that promote psychosis-like behaviors [24]. Our study demonstrates under in vitro and in vivo conditions that chronic THC promotes a functional sensitization of 5-HT2AR responses to the hallucinogenic agonist (±)-DOI. To our knowledge, these data provide the first evidence of a psychosis-like alteration of 5-HT2AR signaling after chronic THC administration. Hallucinogenic effects of 5-HT2AR agonists occurs by activation of inhibitory G-proteins [6, 19]. Thus, the specific alteration of agonist-induced signaling bias of 5-HT2AR observed after THC may explain the higher susceptibility to psychosis-like states. In fact, an increased density of the functional conformation of 5-HT2AR has been demonstrated in brain of schizophrenia patients [10]. On the other hand, THC is able to activate Akt/mTOR pathway [15]. Our results also demonstrate that this signaling pathway is implicated in the chronic THC-induced modulation of 5-HT2AR functionality, since the mTOR inhibitor rapamycin blocked the sensitization.

Sensorimotor gating alterations are recognized as an endophenotype of schizophrenia [25] and loss-of-normal PPI occurs in schizophrenia patients [26]. PPI evaluation shows high-translational validity, as it can be assessed in both rodents and humans [20, 21, 27]. In line with other studies [22, 28], chronic THC per se does not alter sensorimotor gating process. However, decreases [29, 30] or no effects [31, 32] have been reported after chronic synthetic cannabinoids administration (WIN55,212–2 or CP55,940). This discrepancy may be explained because distinct cannabinoid ligands can activate different G-protein subtypes [18]. At this point, as the main psychoactive component and the key player in the psychotomimetic effects of cannabis, the effects of THC should be considered as more representative of the alterations induced by chronic cannabis abuse in humans.

Interactions between the endocannabinoid and serotonergic systems have been widely reported in rodents and recently, a CB1R-5-HT2AR heterodimer has been proposed [33]. However, studies assessing cannabinoid effects on 5-HT2AR functionality are limited. Chronic synthetic cannabinoids seem to potentiate (±)-DOI-mediated responses [34]; whereas, the acute administration of some cannabinoids inhibits (±)-DOI-induced head-twitch responses [35]. On the other hand, the antipsychotic risperidone, a 5-HT2AR inverse agonist, reverses acute THC-induced PPI deficits [36]. Moreover, cannabis-induced psychosis in humans is also responsive to treatment with atypical antipsychotics [37]. Therefore, it seems that psychotic effect induced by acute THC should be differentiated from other modulation processes induced after chronic exposure.

Although disruptions of PPI after acute (±)-DOI administration are widely described [38, 39], our study demonstrates a marked supersensitivity to this disruption after chronic THC. The responses to (±)-DOI become increased together with an enhanced high-affinity conformation of the 5-HT2AR. Classically, the high-affinity conformation of G-protein receptors is assumed as the functionally active state and represents the receptor fraction coupled to G-proteins [40]. Here, the enhanced coupling is selectively observed toward inhibitory G-proteins, which is considered the pro-hallucinogenic signaling pathway of this receptor. Accordingly, no changes were observed in total expression and density of 5-HT2AR. In this context, an upregulation of cortical 5-HT2AR mRNA and protein expression has been observed following a 7-day treatment with the synthetic cannabinoid CP55,940 [41]. However, as we observed in the present study, it has been described that chronic THC (10 mg/kg) do not modulate 5-HT2AR density in mice brain [42]. This fact supports again the use of THC, rather than other cannabinoid ligand, to evaluate the effects of cannabis abuse. The washout period becomes crucial in studies assessing chronic effects of cannabinoids, as they are able to accumulate in adipose tissue and may induce effects due to the residual presence of the drug. Our data about THC brain concentrations reached during treatment strongly support that the observed effects were due to enduring brain changes derived from the chronic exposure to THC.

The present is the first demonstration that chronic THC leads to supersensitive coupling of 5-HT2AR to inhibitory G-proteins whereas Gαq/11-protein signaling pathway remains unaltered. 5-HT2AR canonically activates phospholipase C via Gαq/11-proteins but it can also activate phospholipase A2, as well as other G-protein subtypes, depending on the ligand used [43]. Hallucinogenic and non-hallucinogenic serotonergic drugs activate the same population of 5-HT2AR in cortical pyramidal neurons, but differ in the receptor-dependent pattern of G-protein signaling and gene transcription induction that they elicit. In human and murine cortical neurons, non-hallucinogenic 5-HT2AR agonists induce *c-fos* expression mediated by Gαq/11-protein-dependent phospholipase C activation. In contrast, hallucinogen 5-HT2AR agonists such as (±)-DOI or LSD, acting at the 5-HT2AR, not only activate *c-fos* but also induce *egr-2* expression, which is a Gαi/o-protein-dependent response. This signaling pattern has been proposed as a specific fingerprint of hallucinogenic pharmacological properties [44].

Although further testing is needed, the selective overactivation of inhibitory G-proteins by (±)-DOI after THC treatment provides a crucial aspect regarding how chronic THC exposure could increase vulnerability to psychosis. Remarkably, selective hypersensitive 5-HT2AR coupling to Gαi/o proteins has also been demonstrated in post mortem frontal cortex of schizophrenia patients [45].

Our results also demonstrate the involvement of the Akt/mTOR signaling pathway in the molecular mechanisms underlying the THC-induced modulation of 5-HT2AR. Rapamycin inhibits mTOR complex 1 (mTORC1), a protein complex that plays an important role in protein synthesis, cell-cycle progression, cell growth and proliferation [46, 47]. The protein kinase Akt activates mTORC1 that subsequently activates ribosomal protein S6, and regulates axonal branching [11]. This complex has been involved in THC-induced memory deficits [15]. Moreover, polymorphisms in the *AKT1* gene are known to increase cannabis-induced psychotic responses [48, 49]. Interestingly, ablation of both phospho(Ser473)-Akt and S6 kinase (S6K), whose target substrate is the S6 ribosomal protein, is able to alter 5-HT2AR functionality and signaling [50]. Our data demonstrate that rapamycin prevents the THC-induced activation of Akt/mTOR pathway and restores the alterations in the 5-HT2AR functionality. Nonetheless, the involvement of other signaling pathways, as well as the modulation of other cellular mechanisms induced by the chronic administration of rapamycin cannot be discarded.

In summary, the present study demonstrates that chronic THC exposure leads to the overactivation of a pro-hallucinogenic signaling pathway of 5-HT2AR through the regulation of Akt/mTOR pathway. Findings of the present work describe, for the first time, the molecular mechanisms underlying the link between cannabis abuse and susceptibility to schizophrenia-like symptoms.

## Electronic supplementary material

Supplemental Material
