# Supplementary material for: Chronic cannabis promotes pro-hallucinogenic signaling of 5-HT2A receptors through Akt/mTOR pathway
Source: Neuropsychopharmacology. 2018 Apr 27;43(10):2028–35. doi: 10.1038/s41386-018-0076-y (PMC6098160; doi:10.1038/s41386-018-0076-y)
Supplement: Supplementary file 1 — Supplemental Material [file 41386_2018_76_MOESM1_ESM.docx]

**SUPPLEMENTARY MATERIAL**

**Chronic cannabis promotes pro-hallucinogenic signaling of 5-HT_2A_ receptors through Akt/mTOR pathway**

Inés Ibarra-Lecue B.Sc.^1^, Irene Mollinedo-Gajate Ph.D.^1^, J Javier Meana M.D. Ph.D.^1,2^, Luis F Callado M.D. Ph.D.^1,2^, Rebeca Díez-Alarcia Ph.D.^1,2^ and Leyre Urigüen Ph.D.^1,2^

*^1^Department of Pharmacology, University of the Basque Country UPV/EHU and Centro de Investigación Biomédica en Red de Salud Mental CIBERSAM, Spain*

*^2^Biocruces Health Research Institute, Bizkaia, Spain*

**Content:**

**Supplementary Tables 1-3**

**Supplementary Figures 1-4**

**Supplementary Table 1. Details of the antibodies used and their dilutions.**

| **Antibody type** | **Target** | **Company** | **Reference** | **Dilution** |
| --- | --- | --- | --- | --- |
| Primary | 5-HT_2A_R | Santa Cruz Biotech. | Sc-166775 | 1:20,000 |
| Primary | Akt | Cell Signaling | 9272 | 1:1,000 |
| Primary | Phospho(Ser^473^)‑Akt | Cell Signaling | 4060 | 1:1,000 |
| Primary | rpS6 | Cell Signaling | 2317 | 1:500 |
| Primary | Phospho(Ser^235/236^)‑rpS6 | Cell Signaling | 4858 | 1:500 |
| Primary (rabbit) | β‑actin | Abcam | Ab8227 | 1:20,000 |
| Primary (mouse) | β‑actin | Sigma Aldrich | A1978 | 1:200,000 |
| Secondary | Alexa Fluor 680 anti‑Rabbit Ig-G | Thermo Fisher Sc. | A21076 | 1:2,500 |
| Secondary | Alexa Fluor 680 anti‑Mouse Ig-G | Thermo Fisher Sc. | A21057 | 1:4,000 |
| Secondary | DyLight 800 Anti-Rabbit Ig-G | Rockland Immunoc. | 610-745-127 | 1:5,000 |
| Secondary | DyLight 800 Anti-Mouse Ig-G | Rockland Immunoc. | 610-745-002 | 1:10,000 |

5-HT_2A_R = Serotonin 2A receptor; Ig‑G = Immunoglobulin G; Biotech. = Biotechnology; Sc. = Scientific; Immunoc. = Immunochemicals

**Supplementary Table 2.**

Pharmacological parameters of [^3^H]ketanserin binding displacement curves by (±)‑DOI in brain cortex membrane preparations of THC treated (10 mg/kg, i.p., 30 days) and control (vehicle) mice.


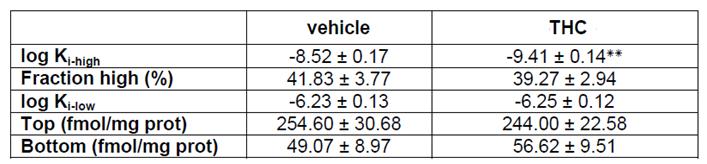


***p*<0.01; unpaired Student’s *t*-test.

Data were best fit to a biphasic compared to monophasic displacement curve by *F*-test (F (2,48)=41.14, *p*<0.0001 for vehicle curves; F (2,42)=73.10, *p*<0.0001 for THC curves).

All values represent means ± SEM of four experiments carried out in duplicates.

**Supplementary Table 3.**

Summary of three-way repeated measures ANOVA analysis performed to test the effects of dB, THC, (±)-DOI and rapamycin as well as the potential interactions in %PPI.

|  | **Source of variation** | **DFn** | **DFd** | **F** | ***p*** |
| --- | --- | --- | --- | --- | --- |
| **Figure 2A** | THC | 1 | 51 | 0.7 | 0.4057 |
|  | **DOI** | **1** | **51** | **38.56** | **<0.001** |
|  | **dB** | **2** | **102** | **118.86** | **<0.001** |
|  | **THC x DOI** | **1** | **51** | **6.48** | **0.0140** |
|  | THC x dB | 2 | 102 | 0.89 | 0.4141 |
|  | DOI x dB | 2 | 102 | 2.00 | 0.1410 |
|  | THC x DOI x dB | 2 | 102 | 1.68 | 0.1909 |
| **Figure 5C** | Rapamycin | 1 | 35 | 1.25 | 0.2719 |
|  | THC | 1 | 35 | 3.57 | 0.0673 |
|  | **dB** | **2** | **70** | **48.03** | **<0.001** |
|  | **Rapamycin x THC** | **1** | **35** | **4.50** | **0.0410** |
|  | Rapamycin x dB | 2 | 70 | 0.09 | 0.9150 |
|  | THC x dB | 2 | 70 | 0.44 | 0.6444 |
|  | Rapamycin x THC x dB | 2 | 70 | 2.32 | 0.1056 |

Significant *p* values are shown in bold.

**Supplementary Figure 1.**

%PPI and startle amplitude of mice treated with two different doses of (±)-DOI (0.25 and 0.5 mg/kg, i.p.).

(**A**) Acute (±)‑DOI exerted similar effects in all the prepulse intensities. Significant effect was only found with (±)‑DOI 0.5 mg/kg (two-way ANOVA: DOI effect; F(2,48)=25.70, *p*<0.0001; n=5 each group). (**B**) Startle amplitude was not significantly modulated. Bonferroni’s *post-hoc* comparisons: **p*<0.05, ***p*<0.01 and ****p*<0.001 *vs*. saline.

**Supplementary Figure 2.**

%PPI and startle amplitude of mice treated with rapamycin (5 mg/kg, 4 days/week, 30 days) or vehicle.

(**A**) Acute (±)‑DOI exerted similar effects on PPI, independently of the pretreatment with vehicle or rapamycin. (two-way ANOVA: DOI effect; 82 dB F(1,51)=6.18; 87 dB F(1,51)=6.30, *p*<0.05; n=7‑16). (**B**) Startle amplitude was not modulated.

**Supplementary Figure 3.**

Immunoreactivity of phospho-Akt (Ser473), total Akt, phospho-rpS6 (Ser235/236) and rpS6 in brain cortical tissue homogenates of the different groups and their ratios for rapamycin-treated and control animals.

(**A**) Phospho-Akt immunoreactivity was increased in cortical tissue of THC‑treated mice, whereas this increase was totally abolished with rapamycin concomitant treatment (two-way ANOVA: rapamycin x THC; F(1,26)=11.67, *p*<0.01; n=7-8). (**B**) No changes on total Akt were observed (n=8 each group). (**C**) Phospho-rpS6 was also increased after chronic THC and blocked with rapamycin (two-way ANOVA: rapamycin x THC; F(1,22)=6.70, *p*<0.05; n=6-7). (**D**) Rapamycin treatment decreased total rpS6 in THC-treated mice (two-way ANOVA: rapamycin x THC; F(1,24)=4.61, *p*<0.05; n=7 each group). (**E**) Ratios of phospho-Akt/Akt and (**F**) phospho-rpS6/rpS6 in cortical tissue of rapamycin-treated mice showed no changes compared with control animals. Bonferroni’s *post-hoc* comparisons: **p*<0.05 and ***p*<0.01 *vs*. vehicle; ###*p*<0.001 *vs*. THC.

**Supplementary Figure 4.**

(±)‑DOI induced [^35^S]GTPγS binding stimulation coupled to immunoprecipitation with specific antibodies against different Gα‑protein subtypes in cortical membranes of mice chronically treated with rapamycin (5 mg/kg, 4 days/week, 30 days) or vehicle.

No differences were found in the stimulation of any Gα‑protein subtype between both groups.
